# Extracellular vesicle‐derived microRNA‐18b ameliorates preeclampsia by enhancing trophoblast proliferation and migration via Notch2/TIM3/mTORC1 axis

**DOI:** 10.1111/jcmm.16234

**Published:** 2021-04-09

**Authors:** Zhongmei Yang, Nan Shan, Qinyin Deng, Yujue Wang, Yan Hou, Jie Mei, Zhao Wu

**Affiliations:** ^1^ Department of Obstetrics and Gynecology Sichuan Academy of Medical Sciences & Sichuan Provincial People's Hospital Chengdu China; ^2^ Department of Obstetrics and Gynecology The First Affiliated Hospital of Chongqing Medical University Chongqing China

**Keywords:** extracellular vesicle, MicroRNA‐18b, migration, notch receptor 2, preeclampsia, proliferation, Rapamycin complex 1, T‐cell immunoglobulin and mucin domain‐containing protein 3, trophoblast

## Abstract

Preeclampsia (PE), a common disorder of pregnancy, is characterized by insufficient trophoblast migration and inadequate vascular remodelling, such that promotion of trophoblast proliferation might ameliorate PE. In the current study, we sought to study the underlying mechanism of extracellular vesicle (EV)‐derived microRNA‐18 (miR‐18b) in PE. Human umbilical cord mesenchymal stem cells (HUCMSCs) isolated from placental tissues were verified through osteogenic, adipogenic and chondrogenic differentiation assays. Bioinformatics analyses and dual‐luciferase reporter gene assay were adopted to confirm the targeting relationship between miR‐18b and Notch2. The functional roles of EV‐derived miR‐18b and Notch2 in trophoblasts were determined using loss‐ and gain‐of‐function experiments, and trophoblast proliferation and migration were assayed using CCK‐8 and Transwell tests. In vivo experiments were conducted to determine the effect of EV‐derived miR‐18b, Notch2 and TIM3/mTORC1 in a rat model of PE, with monitoring of blood pressure and urine proteinuria. TUNEL staining was conducted to observe the cell apoptosis of placental tissues of PE rats. We found down‐regulated miR‐18b expression, and elevated Notch2, TIM3 and mTORC1 levels in the placental tissues of PE patients compared with normal placenta. miR‐18b was delivered to trophoblasts and targeted Notch2 and negatively its expression, whereas Notch2 positively mediated the expression of TIM3/mTORC1. EV‐derived miR‐18b or Notch2 down‐regulation enhanced trophoblast proliferation and migration in vitro and decreased blood pressure and 24 hours urinary protein in PE rats by deactivating the TIM3/mTORC1 axis in vivo. In summary, EV‐derived miR‐18b promoted trophoblast proliferation and migration via down‐regulation of Notch2‐dependent TIM3/mTORC1.

## 1 INTRODUCTION

Preeclampsia (PE), one of the most serious complications of pregnancy, is characterized by initial‐onset hypertension and proteinuria, which can in severe cases lead to the death of mother and foetus.^1^ The trophoblasts are essential elements of the placenta with an ability to invade and differentiate in the decidua and a portion of the myometrium, and mediate remodelling of the maternal vasculature. The occurrence of PE is largely attributed to suppressed trophoblast invasion to the uterus and the consequently impaired remodelling of the maternal spiral artery.^2^ Trophoblast invasion and placental access to the maternal blood supply are necessary factors in normal foetal development, but their inhibition is likely to induce PE.^3^ Elucidating the mechanisms underlying trophoblast proliferation and invasion is therefore a key to understanding and treating PE.

Membrane encapsulated extracellular vesicles (EVs) are secreted into the extracellular environment, where they can affect inflammation, apoptosis and angiogenesis.^4^ EVs derived from human umbilical cord mesenchymal stem cells (HUCMSCs) protected the morphology and angiogenesis of placenta in rats with PE.^5^ microRNAs (miRs) belong to small and endogenous noncoding RNAs, which are engaged in disease progression including PE.^6^ Indeed, EV‐derived miRs in maternal circulation may serve as early biomarkers of PE.^7^ Interestingly, miR‐18b is reported to regulate cell invasion and migration of trophoblasts in PE.^8^ In the current study, our bioinformatics analysis identified notch receptor 2 (Notch2) as a target of miR‐18b. As a Notch receptor, Notch2 expression is generally up‐regulated in various cancers and exhibits tumour‐promoting properties.^9^ Notch2 is also reported to regulate the proliferation and invasion of the percentage of trophoblasts.^10^ A prior study noted that T‐cell immunoglobulin and mucin domain‐containing protein 3 (TIM3) could be targeted by Notch2.^11^ TIM3 functions as a receptor on interferon‐γ‐producing CD4 and CD8 T cells,^12^ and is involved in the pathogenesis of PE.^13^ Thus, we formed a hypothesis that EV‐derived miR‐18b may exert effects on PE via signalling at Notch2‐dependent TIM3. To test this hypothesis, we established mouse model of PE and human trophoblast cell line HTR‐8/SVneo to explore the possible regulatory network, aiming to inform new treatment strategies against PE.

## 2 MATERIAL AND METHODS

### 2.1 Ethical approval

The study was carried out under approval by the Ethics Committee of Sichuan Academy of Medical Sciences & Sichuan Provincial People's Hospital and followed the tenets of the *Declaration of Helsinki*. Written informed consent was obtained from patients or newborns' guardians. Animals were treated humanely using approved procedures in compliance with the recommendations in the Guide for the Care and Use of Laboratory Animals of the National Institutes of Health.

### 2.2 Samples

A total of 20 patients with PE and 20 women with normotensive pregnancy who were treated in the Department of Obstetrics and Gynecology of Sichuan Academy of Medical Sciences & Sichuan Provincial People's Hospital from January 2018 to February 2019 were selected in this study. The average age was (28.50 ± 3.93) years, and the average gestational week was (34.15 ± 3.12) weeks. The patients with PE were recruited during their first pregnancy with systolic blood pressure ≥ 140 mm Hg (1 mm Hg = 0.133 kPa), diastolic blood pressure ≥ 90 mm Hg after 20 weeks of pregnancy and urine protein ≥ 0.3 g/d or random urine protein (+) and the above. After delivery, placental tissues (1 cm × 1 cm × 1 cm) were collected in a sterile environment and washed three times in phosphate buffer saline (PBS). The decidual and placental tissues were carefully removed and the remaining placental tissues were minced to about 1 mm^3^ portions, which were added with 4 mL of medium and cultured in an incubator at 37°C with 5% CO_2_ incubator. Within 24 hours of delivery, 3 mL of venous blood was collected from each woman during fasting and centrifuged at 1509.3 *g* for 15 minutes. The upper layer of serum was collected, centrifuged, and then stored in dry and sterile Eppendorf (EP) tubes at −80°C for subsequent experiments.

### 2.3 Enzyme‐linked immunosorbent assay (ELISA)

The frozen serum samples were thawed at room temperature, and the content of mTORC1 in the serum was measured strictly in accordance with the instructions provided by the ELISA kit (1535421332, Laibio, Shanghai, China). The absorbance (A) of each well at 450 nm was measured using a universal microplate reader (Synergy 2, BioTek, Winooski, VT, USA) within 3 minutes, and a linear standard curve was plotted for calculation of sample concentrations.

### 2.4 Isolation and culture of HUCMSCs

Human umbilical cord blood was obtained from newborns delivered by caesarean and mixed with an equal volume of PBS. After 30 minutes, the mixture was centrifuged at 1006.2 *g* for 10 minutes at room temperature, and the supernatant was stored at −20°C for 1 month prior to transfer to a −80°C freezer for subsequent experiments. The umbilical cord blood was mixed with PBS of an equal volume, followed by dilution. The thawed blood samples diluted in equal volumes of 1:1 were added in Ficoll‐Hypaque solution (P7794, Sigma) and centrifuged at 2500 rpm for 20 minutes at room temperature. The white interface cell layer was aspirated and centrifuged at 800 rpm for 5 minutes to obtain suspension of HUCMSCs.

### 2.5 Flow cytometry

Suspended cells were seeded in a T‐25 cm^2^ cell culture flask at 1 × 10^6^ cells/cm^2^ and cultured at 37°C in 5% CO_2_. After three days, the non‐adherent cells were removed after the medium was aspirated. When cell confluence reached 80%‐90%, cells were trypsinized (0.25%), passaged and cultured in a constant temperature incubator at 37°C in 5% CO_2_. Human umbilical cord mesenchymal stem cells were trypsinized (0.25 g/dl) for 5 minutes and centrifuged at 6037.2 *g* for 5 minutes. The pellet was collected and added with phycoerythrin‐labelled mouse anti‐human CD14, CD31, CD34, CD45, CD166 or fluorescein isothiocyanate (FITC)‐labelled CD29, CD44, HLA‐DR monoclonal antibodies, with PBS serving as control. The samples were incubated at room temperature for 30 minutes in the dark and added with FITC‐labelled secondary antibody goat anti‐mouse immunoglobulin G (IgG) for a further 15 minutes incubation in the dark, and then placed on ice for determination by a flow cytometry.

### 2.6 Osteogenic, adipogenic and chondrogenic differentiation of HUCMSCs

Human umbilical cord mesenchymal stem cells were inoculated into 6‐well plate (5 × 10^5^ cells/well) and cultured in complete medium. Upon reaching 80%‐90% cell confluence, the medium was replaced with osteogenic induction solution (10 nmol/L β‐glycerophosphate, 50 μmol l‐vitamin C, 5 mmol/L FK506, 100 nmol/L dexamethasone). The solution was renewed every three days, and the morphological changes were observed under a phase‐contrast microscope (ckx41‐a32ph, Pooher, Shanghai, China) every day. On the 21st day of osteogenic induction culture, after the cell culture medium was removed, cells were fixed with 4% polyformaldehyde at room temperature for 20 minutes and added with 40 mm alizarin red for 10 minutes.

The adipogenic differentiation of HUCMSCs was conducted as previously performed. In brief, the medium was replaced with adipogenic induction solution (1 μmol/L dexamethasone, 10 mg/L recombinant human insulin, 0.5 mmol/L isobutylmethylxanthine). On days 7‐10 of adipogenic induction, the round‐shaped transparent lipid droplets were observed and on the 28th day of adipogenic induction culture, cells were stained with oil red O for 30 minutes prior to microscopic examination.

The chondrogenic differentiation of HUCMSCs was conducted as previously. In brief, the medium was replaced with chondrogenic induction solution (0.1 mmol/L l‐vitamin C, 1 mmol/L sodium pyruvate, 10 ng/mL transforming growth factor beta 3, 0.1 nmol/L dexamethasone, 6.25 μg/mL transferrin, 6.25 μg/mL recombinant human insulin, 1.25 ng/mL bovine serum albumin, 6.25 ng/mL selenite, 5.35 μg/mL linoleic acid). On the 16th day of chondrogenic induction, the cells were immersed in 0.1 N HCl solution for 5 minutes to reduce the pH to 1.0, whereupon the cells were added with alcian blue for staining overnight. The next morning, the cells were rinsed three times in 0.1 N HCl solution for 5 minutes and then photographed under a phase‐contrast microscope.

### 2.7 EV isolation

Human umbilical cord mesenchymal stem cells were seeded into EV‐free Roswell Park Memorial Institute (RPMI) 1640 medium containing 10% foetal bovine serum (FBS) and cultured in a cell incubator at 37°C with 5% CO_2_. The cell supernatant was collected two days later, and the cell mass was removed by a 0.22 μm filter. The supernatant was taken following centrifugation at 300 rpm for 10 minutes at 4°C, and dead cells were removed by centrifugation at 3000 rpm for 10 minutes followed by recentrifugation at 5 031 *g* for 30 minutes to remove cell debris. The supernatant was ultracentrifuged at 110 000 rpm for 40 minutes, and the 5 mL of liquid at the bottom of the centrifuge tube was isolated and resuspended. This liquid was centrifuged again at 4°C, and 110 000 rpm for 90 minutes, and the pellet resuspended in 10 mL PBS for a final centrifugation at 4°C and 55 341 *g* for 90 minutes. The isolated EVs were finally resuspended in 100 μL PBS and stored at −80°C.

### 2.8 EV evaluation

The EVs were precipitated and immediately fixed in 2.5% glutaraldehyde at 4°C, followed by dehydration in gradient alcohol and immersion in epoxy resin for embedding by standard procedures. Ultra‐thin sections were stained with uranyl acetate and lead citrate and observed under a transmission electron microscope (TEM) (JEM‐1010, JEOL, Tokyo, Japan).

EVs were resuspended with 25 μL of PBS and added with phycoerythrin‐labelled mouse anti‐human CD63, CD81, CD166 or allophycocyanin‐labelled CD73, CD90 5 μL. PBS served as primary antibody for control, incubated for 30 minutes and centrifuged at 10 000 rpm for 15 minutes. The supernatant was discarded, EVs were added with 200 μL PBS and placed on ice for determination using flow cytometry. Nanoparticle Tracking Analysis (NTA) was performed to evaluate EVs. In brief, the collected EVs were diluted with PBS at a concentration of 10^6^‐10^9^ cells/mL, and then, a sample was drawn with a 1 mL syringe and injected into a Nanosight analyser (Nanosight NS300, Malvern, UK) for detection and analysis.

### 2.9 Establishment of a rat model of PE

Adult Sprague‐Dawley rats (purchased from Nanjing Junke Biological Co., LTD), aged 14‐16 weeks old, consisting of 75 male (210‐260 g) and 75 female rats (190‐230 g), were held in a colony with temperature of 18‐28°C with relative humidity of 40%‐70% and free access to food and water. Then, male and female rats during oestrus were caged together at a ratio of 1:2. The first day of pregnancy was confirmed by the presence of sperm. Pregnant rats were injected subcutaneously with NG‐Nitro‐L‐arginine Methyl Ester (125 mg/d), and a rat model of PE was established after drug administration for seven days.^14^ Forty rats with the PE model were selected at random and assigned to negative control for overexpression vector (oe‐NC), oe‐Notch2, NC for short hairpin RNA (sh‐NC), sh‐Notch2, NC, TIM3, oe‐Notch + NC or oe‐Notch + TIM3 groups (five rats/group).

### 2.10 Blood pressure and urine protein concentration

Groups of rats with PE without plasmid transfection were treated with EVs isolated from cord blood of PE patients: Normal group, Sham group, PE group, PE + NC group (PBS instead of EVs), PE + L‐EV group (EV concentration: 50 mg/(kg/d)), PE + M‐EV group (EV concentration: 75 mg/(kg/d)), PE + H‐EV group (EV concentration: 100 mg/(kg/d)) (five rats/group). A non‐invasive blood pressure heart rate meter (DSWY‐1, Instrument Factory) was adopted to measure blood pressure with averaging of three recordings within 5 minutes. The rats were placed in a metabolic cage with normal feeding. The 24‐hour urine was collected, urine volume was measured, and urine protein concentration was determined by a fully automatic biochemical analyser (PUZS‐300X, Perlong).

### 2.11 Terminal deoxyribonucleotidyl transferase‐mediated deoxyuridine‐5′‐triphosphate Nick‐End Labelling (TUNEL)

Sections were added with 50 μL of 1% proteinase K diluent and incubated at 37°C for 30 minutes. The sections were added with 0.3% H_2_O_2_ methanol to eliminate endogenous peroxidase (POD) activity, incubated for 30 minutes at room temperature, added with TUNEL reaction solution and incubated in a wet box at 37°C for 1 hour. The sections were added with 50 μL Converter‐POD, 2% diaminobenzine solution and incubated at room temperature for 15 minutes. After microscopic examination revealed brown‐yellow nuclear staining, the reaction was terminated by adding distilled water, followed by haematoxylin counterstaining, and dehydration using gradient ethanol (50%, 70%, 90% and 100%). The sections were cleared with xylene, mounted using a neutral gum and observed under an optical microscope. Each section was scored in five randomly selected fields of view, where the positive and negative cells were counted. The cells with brown nuclei were positive cells (apoptotic cells), while cells with blue nucleus were normal cells. The apoptotic index was calculated by the number of positive cells/the number of negative cells.

### 2.12 Cell transfection and grouping

HTR‐8/SVneo cell lines were divided into the miR‐18b mimic, NC mimic, miR‐18b inhibitor, NC inhibitor, Notch2 mimic (Notch2) or Notch2 NC (Vector) groups. In brief, one day before transfection, HTR‐8/SVneo cells were seeded in a 12‐well cell culture plate at a concentration of 1 × 10^5^ cells/mL. Upon attaining 50%‐70% cell confluence, the cells were added with 800 μL of serum‐free medium, and the above‐mentioned plasmid and lipo2000 mixed solution (Cat. No. 11668027, Thermo Fisher Scientific, USA) were added to a 12‐well plate respectively. The medium was renewed after 6 hour of cell culture and cells were collected 48 hour after transfection.

### 2.13 Co‐culture of EVs and trophoblasts

EVs were co‐cultured with trophoblasts and subgrouped as follows: PE‐EV^agomir‐NC^ (EVs containing agomir‐NC co‐cultured with trophoblasts ), PE‐EV^miR‐18b agomir^ (EVs containing miR‐18b agomir co‐cultured with trophoblasts), PE‐EV^antagomir‐NC^ (EVs containing antagomir‐NC co‐cultured with trophoblasts), PE‐EV^miR‐18b^ antagomir (EVs containing miR‐18b antagomir co‐cultured with trophoblasts), PE‐EV^NC^ (EV‐containing NC co‐cultured with trophoblasts), PE‐EV^GW4869^ (EVs containing GW4869 co‐cultured with trophoblasts), PE‐EV^miR‐18b^ + Vector (EV‐containing miR‐18b agomir co‐cultured with trophoblasts transfected with Vector), PE‐EV^miR‐18b^ + Notch2 (EVs containing miR‐18b agomir co‐cultured with trophoblasts transfected with Notch2). HTR‐8/SVneo cells were cultured in the serum‐deprived medium for 12 hours. HTR‐8/SVneo cells were plated at 6 × 10^5^ cells/well in 6‐well plates containing 5% FBS in RPMI 1640 medium. Extracellular vesicles extracted from transfected PE rats were co‐cultured with HTR‐8/SVneo cells at a concentration of 100 μg/mL for three days.

### 2.14 Immunofluorescence

Extracellular vesicle cell suspension (100 μL) was added with PKH26 staining working solution, mixed by pipetting, and fixed at room temperature for 1 minute. Cell suspension was added with PBS to a final volume of 7 mL and centrifuged at 120 000 *g* for 70 minutes before the supernatants were discarded. EVs were resuspended in 500 μL of complete medium and added with 100 μL of EVs to a 3.5 cm co‐culture dish with trophoblasts, followed by incubation for 4 hours at 37°C in a 5% CO_2_ incubator. The cell culture was aspirated, and the cells were fixed with 4% paraformaldehyde at room temperature and permeabilized with 0.1% Triton working solution for 10 minutes. After aspiration of the working solution, the cells were blocked with 5% bovine serum albumin (BSA) for 60 minutes and added with primary antibody and incubated at 2‐8°C overnight. Then the sections were mounted with 4′, 6‐diamidino‐2‐phenylindole (DAPI), followed by incubation for 5 minutes in the dark prior to observation under a laser confocal microscope.

### 2.15 Bioinformatics analyses

The downstream target genes of miR were predicted using the database starBase (clipExpNum > 5, pancancerNum > 5) (http://starbase.sysu.edu.cn/), mirDIP (Integrated Score > 0.6) (http://ophid.utoronto.ca/mirDIP/), RAID (Score > 0.6) (http://www.rna‐society.org/raid2/index.html), microRNA (energy < −20) (http://www.microrna.org/microrna/home.do) and TargetScan (Cumulative weighted context ++ score < −0.1) (http://www.targetscan.org/vert_71/). The Venn plot was mapped to identify the most relevant genes, and a protein‐protein interaction (PPI) network map was constructed using GeneMANIA (http://genemania.org), followed by Cytoscape (https://cytoscape.org) to identify the most critical downstream gene. Downstream regulatory pathway and co‐expression analysis were conducted using MEM (https://biit.cs.ut.ee/mem/index.cgi).

### 2.16 Dual‐luciferase reporter gene assay

The 293T cells were purchased from the Cell Bank of Chinese Academy of Sciences (Shanghai, China) and cultured in Dulbecco's Modified Eagle's Medium. Upon attaining 80%‐90% cell confluence, cells were trypsinized (0.25%), passaged and cultured in a 5% CO_2_ and 37°C incubator. The dual‐luciferase reporter gene assay was adopted to the targeting relationship between miR‐18b and Notch2. The Notch2 3′untranslated region (3**′**UTR) gene fragment was artificially synthesized and introduced into pmirGLO vector (Promega) using an endonuclease site. The complementary sequence mutant site (MUT) of the seed sequence was designed on the Notch2 wild type (WT). After restriction enzyme digestion, the target fragment was inserted into pGL3‐control vector using T4 DNA ligase. The sequenced luciferase reporter plasmids WT and MUT were co‐transfected with miR‐18b mimic into HEK‐293T cells (Shanghai Cell Bank of Chinese Academy of Sciences), respectively. Cells were collected and lysed 48 hours after transfection, and luciferase activity was determined using the dual‐Luciferase Reporter Assay System kit (Promega) on a Luminometer TD‐20/20 detector (E5311, Promega).

### 2.17 Reverse transcription quantitative polymerase chain reaction (RT‐qPCR)

Total RNA of PE embryonic tissues or cells was extracted following instructions in the Trizol kit (Invitrogen). Contaminated DNA was treated with RNase‐free DNase I, extracted with phenol and chloroform, and the RNA was dissolved in ultra‐pure water treated with diethylpyrocarbonate. The ND‐1000 ultraviolet/visible spectrophotometry Meter (Nanodrop, Rockland, DE, USA) was adopted to measure the absorbance at 260 and 280 nm, and the concentration of total RNA was determined. cDNA was synthesized using a reverse transcription kit, and RT‐qPCR was performed on the synthetized cDNA. U6 served as an internal reference for miR‐18b and β‐actin for the remaining mRNAs. Primers are listed in Table 1. The relative transcription level of the target gene mRNA was calculated by the 2^−△△^Ct method.

**TABLE 1:** Primer sequences for RT‐qPCR

Target gene	Primer sequences
miR‐18b	F: 5′‐GAAAGAAGGCGAGGAGCAGATCGAGGAA‐3′
	R: 5′‐GCGGTAAGGTGCATCTAGTG‐3′
Notch2	F: 5′‐CACAGGAGAGGACTGCCAATACT‐3′
	R: 5′‐TGTCCCGGCTGAGCATGT‐3′
TIM3	F: 5′‐TGCTGCTGCTGCTACTACTTACA‐3′
	R: 5′‐AGGTTGGCCAAAGAGATGAG‐3′
Rheb1	F: 5′‐GCCCAGAACATCTGTTCCAT‐3′
	R: 5′‐GGTACCCACAACCTGACACC‐3′
Rhebl1	F: 5′‐GGAAAGAAGCTGGCAGAGTCCTG‐3′
	R: 5′‐CTAGAGGCCAGTGTCCATGAGAG‐3′
β‐actin	F: 5′‐AGTGTGACGTGGACATCCGCAAAG‐3′
	R: 5′‐ATCCACATCTGCTGGAAGGTGGAC‐3′
U6	F: 5′‐GCTTCGGCAGCACATATACTAAAAT‐3′
	R: 5′‐CGCTTCACGAATTTGAGTGTCAT‐3′

Abbreviations: F, Forward; miR‐18b, MicroRNA‐18b; Notch2, Notch receptor 2; R, Reverse; RT‐qPCR, reverse transcription quantitative polymerase chain reaction; TIM3, T cell immunoglobulin and mucin domain‐containing protein 3.

### 2.18 Immunoblotting

Total protein of PE embryonic tissues, EVs or transfected cells was extracted, and the bicinchoninic acid protein quantitative kit (ThermoFisher Scientific) was employed to determine protein concentration. Protein was electrophoretic separated by 10% sodium dodecyl sulphate polyacrylamide gels and transferred to nitrocellulose membranes (ZeYE Biological). The membrane was blocked with 5% skim milk powder and then placed in a 3% BSA blocking buffer. Then, membrane was probed with primary rabbit antibodies to Notch2 (1:1000, ab118824, Abcam), TIM3 (1:500, ab185703, Abcam), mTORC1 (1:1000, ab25880, Abcam), CD9 (1:2000, ab92726, Abcam), CD63 (1:1000, ab134045, Abcam), Calnexin (1:20 000, ab92537, Abcam) and β‐actin (1:1000, ab8227, Abcam), and then re‐probed with IgG antibody complexed to horseradish peroxidase (1:2000, ab97051, Abcam). Enhanced chemiluminescence solution (ECL808‐25, Biomiga) was added on membrane for colour development. After scanning and with an optical luminometer (GE, Pittsburg, USA), the protein bands were analysed by Image Pro Plus 6.0 software (Media Cybernetics, Silver Spring, Maryland, USA) to analyse the relative expression levels of proteins with β‐actin as the internal reference.

### 2.19 Flow cytometry

UltraComp ebeads (1 μL; Thermo Fisher Scientific) was added in an EP tube containing 300 μL of morpholine ethanesulfonic acid buffer (MES buffer), mixed thoroughly and centrifuged at 3000 rpm for 5 minutes. The supernatant was aspirated and discarded. The washed beads were resuspended in 300 μL of MES buffer. After addition of the PE‐EVs suspension, the mixture was left to stand at room temperature for 1 hour. The EP tube was then placed on a shaking table at 4°C overnight. On the next day, the EP tube was centrifuged at low speed for 1 minutes, followed by addition of 1 mmol/L glycine to a final concentration of 200 μmol/L. This mixture was incubated at room temperature for 30 minutes. The beads were resupended in 300 μL of 3% FBS‐MES buffer, and an appropriate amount of relevant antibodies were added, followed by incubation for 40 minutes. The beads were washed twice with 300 μL of 3% FBS‐MES buffer and centrifuged at 3000 rpm for 5 minutes, and the cells resuspended in 250 μL of PBS for flow cytometry.

### 2.20 Transwell assay

Matrigel gel (YB356234, Shanghai Yubio, Shanghai, China) was thawed overnight at 4°C. Cells were detached and counted, and a cell suspension was prepared with complete medium. The cell suspension (1 × 10^4^ cells/μL) was added to the upper chamber, while 800 μL conditioned medium supplemented with 20% FBS was added to the lower chamber, followed by incubation at 37°C for 16 hours. The Transwell plate was removed, dipped in formaldehyde for 10 minutes, stained with 0.1% crystal violet and finally left to sit at room temperature for 30 minutes. Next, the cells on the upper surface were wiped using a cotton swab, and 24 hours later the remaining cells were photographed and counted under an inverted microscope.

### 2.21 Cell Counting Kit‐8 (CCK‐8)

The transfected HTR‐8/SVneo cells were detached and resuspended. The cell concentration was adjusted to 1 × 10^5^ cells/mL and cells were seeded into 96‐well plates at 100 μL/well and cultured overnight at 37°C. The cells were treated according to the instructions of the CCK‐8 kit (Beyotime, Shanghai, China). A total of 10 μL CCK‐8 was added to each well, followed by incubation at 37°C for 1.5 hours. The absorbance of each well at 450 nm was then measured using a microplate reader.

### 2.22 Statistical analysis

SPSS 21.0 (IBM Corp) was applied for data analysis. The measurement data were expressed as mean ± standard deviation of three independent tests. The independent sample *t* test was conducted for the comparison of normally distributed data between two groups. Comparisons among multiple groups were conducted using one‐way analysis of variance (ANOVA), followed by Turkey's post hoc test. Variables were analysed at different time points using Bonferroni‐corrected repeated measures ANOVA *P* < .05 was considered as statistically significance.

## 3 RESULTS

### 3.1 miR‐18b is down‐regulated in PE

**FIGURE 1:**
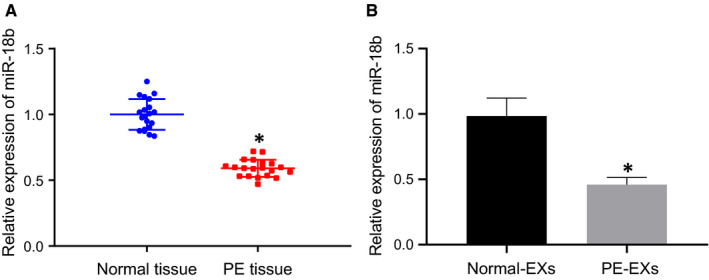
miR‐18b expression is down‐regulated in PE. A, miR‐18b expression in the placental tissues of PE patients or normal placental tissues determined by RT‐qPCR (n = 20). B, miR‐18b expression in the HUCMSCs of PE patients or EVs derived from normal HUCMSCs determined by RT‐qPCR. *P < .05 vs normal placental tissues or normal HUCMSC‐derived EVs. The independent sample *t* test was conducted for the comparison of normally distributed data between two groups

The protective role of HUCMSC‐derived EVs in PE has been noted in a prior study,^5^ while miR‐18b expression is correlated with the development of PE.^8^ To better understand the role of miR‐18b in PE, we adopted RT‐qPCR (Figure 1A,B), which showed that the expression of miR‐18b was remarkably reduced in the placental tissues of PE patients compared to normal placental tissues. Moreover, the expression of miR‐18b in EVs isolated from HUCMSCs of PE patients was markedly lower than that from normal HUCMSCs. Coherently, miR‐18b expression was down‐regulated in placental tissues and HUCMSC‐derived EVs from PE patients.

### 3.2 Successful evaluation of HUCMSCs and HUCMSC‐derived EVs

We isolated HUCMSCs and adopted flow cytometry (Figure S1A), which revealed higher levels of adhesion molecules CD44 and CD29, and low expression of CD106 in the HUCMSCs in the absence of hematopoietic stem cell surface markers (D14, CD34 and CD45), endothelial cell marker (CD31) as well as HLA‐DR. Subsequently, we induced the osteogenesis and differentiation of HUCMSCs (Figure S1B‐D), which provoked the HUCMSCs to cover the bottom of the dish, with uniformly distributed growth in the spindle, and fibroblast‐like shape. In addition, HUCMSCs were fused in vortices or streams‐like with regular arrangement. Subsequently, alizarin red staining revealed mineralized nodule formation and Oil Red O staining revealed the presence of irregularly shaped orange‐yellow lipid droplets. Alcian blue staining revealed that the long spindle‐shaped cells changed to polygonal‐shaped cells, with edges stained blue, all of which consistently indicated the successful osteogenic differentiation of HUCMSCs.

In the subsequent experiments, we evaluated EVs isolated from MSCs. Under TEM (Figure S1E), we observed round or oval‐shaped small vesicles with obvious heterogeneity in size. A membrane structure was observed on the periphery of the vesicles, with a low electron density in the centre. The results of NTA (Figure S1F) displayed that EVs exhibited irregular Brownian motion and had a diameter ranging between 40 and 100 nm. Flow cytometry results showed (Figure S1G) that the common markers for EVs (CD63, CD81) and surface adhesion molecules (CD166, CD73 and CD90) were expressed in MSC‐derived EVs. Immunoblotting results (Figure S1H) displayed that EV‐associated proteins (CD9 and CD63) were poorly expressed, and the EV‐negative protein Calnexin was not expressed in MSC‐derived EVs, thus indicating that MSC‐derived EVs were successfully isolated.

### 3.3 MSC‐derived EVs inhibit PE

**FIGURE 2:**
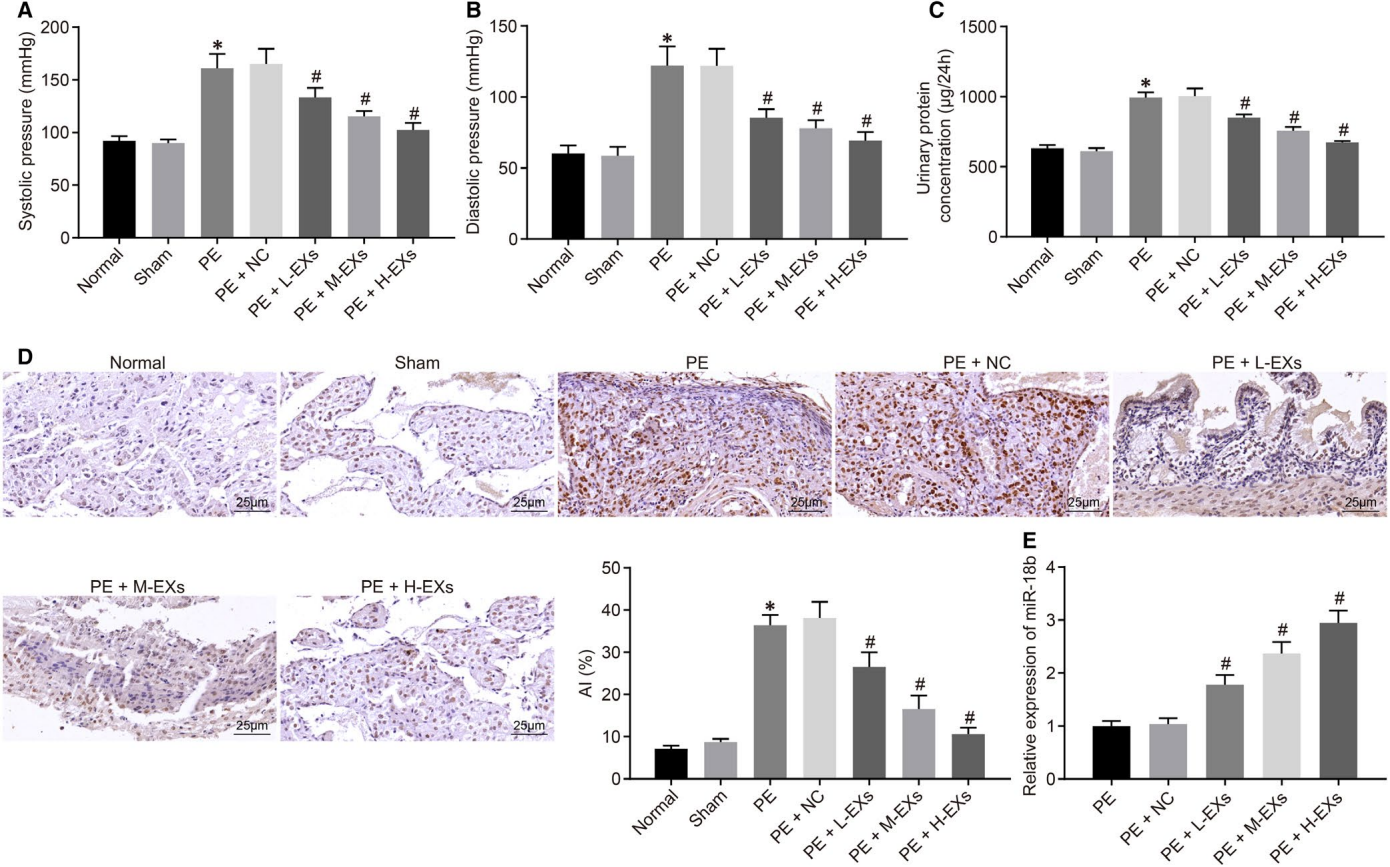
MSC‐derived EVs prevent PE. A, The systolic blood pressure of rats (n = 5/group). B, The diastolic blood pressure of rats. C, The 24‐h urinary protein concentration of rats. D, The cell apoptotic rate determined using TUNEL (×400). E, The expression of miR‐18b in rats. *P < .05 vs sham‐operated rats; #*P* < .05 vs the PE + NC group. Comparisons among multiple groups were conducted using one‐way ANOVA, followed by Turkey's post hoc test

To study the effect of EVs on PE, we constructed a rat model of PE, and then, treated the PE rats with HUCMSC‐derived EVs at different concentrations, with subsequent monitoring of blood pressure and urine protein. TUNEL was adopted to detect the cell apoptosis of placental tissues of rats in different groups (Figure 2A‐D). Results displayed, that compared with normal animals, the systolic blood pressure, diastolic blood pressure, 24‐hour urinary protein concentration, and apoptotic rate in the placental tissues was unaffected in the sham‐operated rats. However, these markers were significantly elevated in the PE group, but were unchanged in the PE + NC rats. These markers all remarkably reduced in the PE + L/M/H‐EVs group, with findings consistent with higher EV concentration. RT‐qPCR results (Figure 2E) showed that, compared with the PE group, the expression of miR‐18b in the PE + NC group remained unchanged. The expression level of miR‐18b in the PE + L/M/H‐EV group gradually increased with treatment with higher EV concentrations. Overall, these results indicated that MSC‐derived EVs could inhibit PE.

### 3.4 miR‐18b derived from MSC‐derived EVs can be delivered to trophoblasts

**FIGURE 3:**
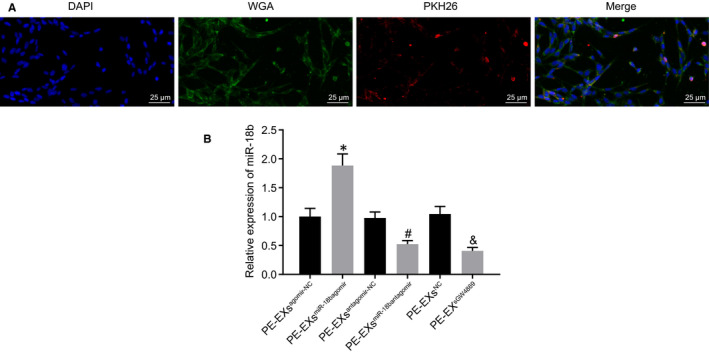
miR‐18b encapsulated in MSC‐derived EVs can be delivered to trophoblasts. A, The EV uptake by HTR‐8/SVneo cells using laser confocal microscope (blue: DAPI‐stained nuclei; green: WGA‐labelled cytoplasm; red: PKH26‐labelled EVs) (×400). B, The expression of miR‐18b in trophoblasts after co‐culture with EVs determined using RT‐qPCR. *P < .05 vs the PE‐EV^agomir‐NC^ group; #*P* < .05 vs the PE‐EV^antagomir‐NC^ group; & *P* < .05 vs PE‐EV^NC^ group. Comparisons among multiple groups were conducted using one‐way ANOVA, followed by Turkey's post hoc test

To study the mechanism of MSCs on trophoblasts, we co‐cultured MSC‐derived EVs with HTR‐8/SVneo cells and detected the EV uptake by HTR‐8/SVneo cells using laser confocal microscopy. This revealed that EVs were taken up by HTR‐8/SVneo cells (Figure 3A). RT‐qPCR results (Figure 3B) confirmed the infection efficiency of the PE‐EV^agomir‐NC^, PE‐EV^miR‐18b agomir^, PE‐EV ^antagomir‐NC^ or PE‐EV^miR‐18b antagomir^ groups. The expression of miR‐18b markedly decreased in the PE‐EV^GW4869^ group. Taken together, miR‐18b derived from MSC‐derived EVs was delivered to trophoblasts.

### 3.5 miR‐18b elevation promotes the proliferation and migration of trophoblasts

**FIGURE 4:**
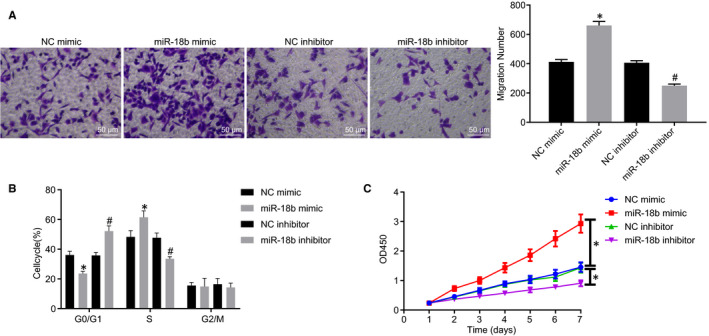
miR‐18b up‐regulation enhances the proliferation and migration of trophoblasts in the NC mimic, miR‐18b mimic, miR‐18b inhibitor or NC inhibitor group. A, The migration of trophoblasts determined by Transwell assay (×200). B, The cell cycle of trophoblasts determined by flow cytometry. C, The proliferation of trophoblasts determined by CCK‐8 assay. *P < .05 vs the NC mimic group; #*P* < .05 vs the NC inhibitor group. Comparisons among multiple groups were conducted using one‐way ANOVA, followed by Turkey's post hoc test. Variables were analysed at different time points using Bonferroni‐corrected repeated measures ANOVA

To elucidate the role of miR‐18b in the proliferation and migration ability of trophoblasts, we transfected HTR‐8/SVneo cells and adopted Transwell, flow cytometry and CCK‐8 assays to determine cell migration, cell cycle and cell proliferation, respectively. As reflected by Figure 4A‐C, compared with the NC mimic group, the HTR‐8/SVneo cells had enhanced migration and proliferation but the miR‐18b mimic group had fewer G0/G1 phase‐arrested as well as more S phase‐arrested cells, whereas opposite effects were noted in the miR‐18b inhibitor group compared to that in the NC inhibitor group. Consistently, these results showed that elevated miR‐18b expression promoted the proliferation and migration of trophoblasts.

### 3.6 miR‐18b inversely targets Notch2

**FIGURE 5:**
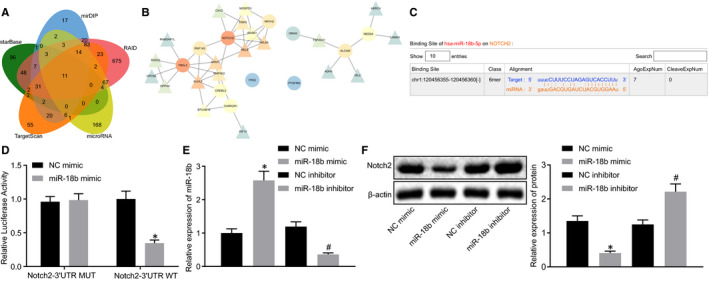
miR‐18b could inversely target Notch2. A, The Venn plot of the downstream target genes of miR‐18b predicted by starBase, mirDIP, RAID, microRNA and TargetScan. B, A PPI network of intersection genes. The circle indicates that the gene is an input intersection gene, while the triangle indicates that the gene is a predicted related gene. The redder indicates higher core degree, which the bluer circle represents lower core degree. C, The predicted binding sites between miR‐18b and Notch2 predicted using starBase. D, The binding relationship of miR‐18b and Notch2 determined using dual‐luciferase reporter gene assay. E, The transfection efficiency of miR‐18b verified using RT‐qPCR. F, The protein level of Notch2 in the HTR‐8/SVneo cells determined using immunoblotting. *P < .05 vs the NC mimic group; #*P* < .05 vs the NC inhibitor group. The independent sample *t* test was conducted for the comparison of normally distributed data between two groups. Comparisons among multiple groups were conducted using one‐way ANOVA, followed by Turkey's post hoc test

To elucidate better the underlying mechanism of miR‐18b, we adopted starBase, mirDIP, RAID, microRNA and TargetScan to predict the downstream target genes of miR‐18b. Results identified 207, 220, 993, 281 and 252 genes, respectively, which were then intersected to obtain 11 overlapped intersection genes (Figure 5A). GeneMANIA was adopted to predict the related genes of these 11 genes and a PPI network was constructed. Cytoscape identified Notch2 as the most core gene in the PPI network (Figure 5B, Table 2). In the subsequent experiment, starBase predicted that miR‐18b targeted Notch2 and indicated a specific binding region between the Notch2 gene and miR‐18b sequences (Figure 5C). Dual‐luciferase reporter gene assay was adopted to verify their binding relationship. As shown in Figure 5D, results revealed that, compared with the NC mimic group, the luciferase activity of WT‐miR‐18b/Notch2 co‐transfected in the miR‐18b mimic group decreased (*P* < .05), while the luciferase activity of the MUT 3**′**UTR remained unchanged (*P* > .05), indicating that miR‐18b could specifically bind to the Notch2 gene.

**TABLE 2:** A PPI network of intersection genes targeted by miR‐18b

Rank	Gene	Degree	Sum weight
1	NOTCH2	8	0.174068927
2	FBXL3	8	0.147579976
3	ALCAM	4	0.099161348
4	NR1H2	4	0.071977937
5	RNF145	4	0.06176728
6	NEDD4	3	0.079360173
7	CAMK2N1	3	0.070694684
8	CREBL2	3	0.055707081
9	ORAI3	1	0.015302694
10	FRS2	0	0
11	PTGFRN	0	0

Note

Degree represents the number of interactions between genes and other genes, while the sum weight represents the scores of sum weight genes and other genes.

Moreover, transfection efficiency of miR‐18b was verified using RT‐qPCR (Figure 5E). Immunoblotting analysis results (Figure 5F) displayed that the protein level of Notch2 was markedly reduced in the miR‐18b mimic group compared to the NC mimic group, but opposite effects were observed in the miR‐18b inhibitor group compared to the NC inhibitor group, suggesting that miR‐18b inhibits Notch2 activity. Taken together, we find that miR‐18b could inversely target Notch2.

### 3.7 miR‐18b promotes the proliferation and migration of trophoblasts by inhibiting Notch2 expression

**FIGURE 6:**
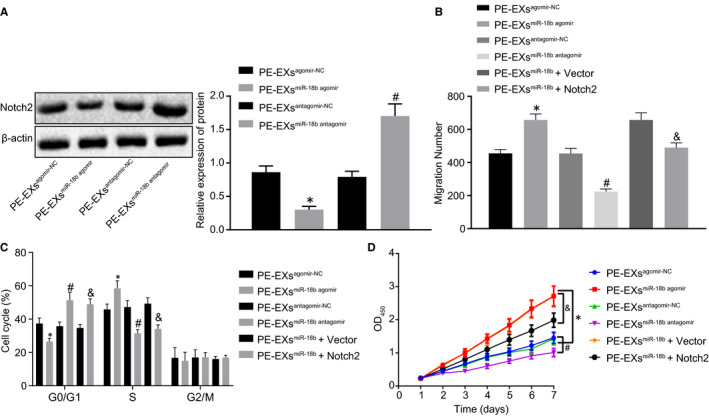
miR‐18b down‐regulates Notch2 expression, thus promoting the proliferation and migration of trophoblasts in PE‐EV^agomir‐NC^, PE‐EV^miR‐18b agomir^, PE‐EV ^antagomir‐NC^ or PE‐EV^miR‐18b antagomir^, PE‐EV^miR‐18b^ + Notch2, PE‐EV^miR‐18b^ + Vector groups. A, The expression of Notch2 after co‐culture determined using immunoblotting. B, The migration of trophoblasts determined by Transwell assay (×200). C, The cell cycle of trophoblasts determined by flow cytometry. D, The proliferation of trophoblasts determined by CCK‐8 assay. *P < .05 vs the PE‐EV^agomir‐NC^ group; #*P* < .05 vs the PE‐EV^antagomir‐NC^ group; & *P* < .05 vs the PE‐EV^miR‐18b^ + Vector group. Comparisons among multiple groups were conducted using one‐way ANOVA, followed by Turkey's post hoc test. Variables were analysed at different time points using Bonferroni‐corrected repeated measures ANOVA

To study the effect of EV‐miR‐18b on trophoblasts, we co‐cultured MSCs‐derived EV with HTR‐8/SVneo cells. Immunoblotting results (Figure 6A) revealed that level of Notch2 was significantly reduced in the PE‐EV^miR‐18b agomir^ group compared with the PE‐EV^agomir‐NC^ group, but opposite results were observed in the PE‐EV^miR‐18b antagomir^ group compared with the PE‐EV^antagomir‐NC^ group. Transwell, flow cytometry and CCK‐8 assays results (Figure 6B‐D) showed the enhanced migration and proliferation of HTR‐8/SVneo cells, but fewer cells in G0/G1 phase as well as more cells in S phase were observed in the PE‐EV^miR‐18b agomir^ group compared with the PE‐EV^agomir‐NC^ group, but opposite findings were observed in the PE‐EV^miR‐18b antagomir^ group compared with the PE‐EV^antagomir‐NC^ group, and a similar trend was detected in the PE‐EV^miR‐18b^ + Notch2 group compared with the PE‐EV^miR‐18b^ + Vector group. Coherently, miR‐18b inhibits Notch2 expression, thereby promoting the proliferation and migration of trophoblasts.

### 3.8 Notch2 exacerbates PE by activating the TIM3/mTORC1 signalling pathway

**FIGURE 7:**
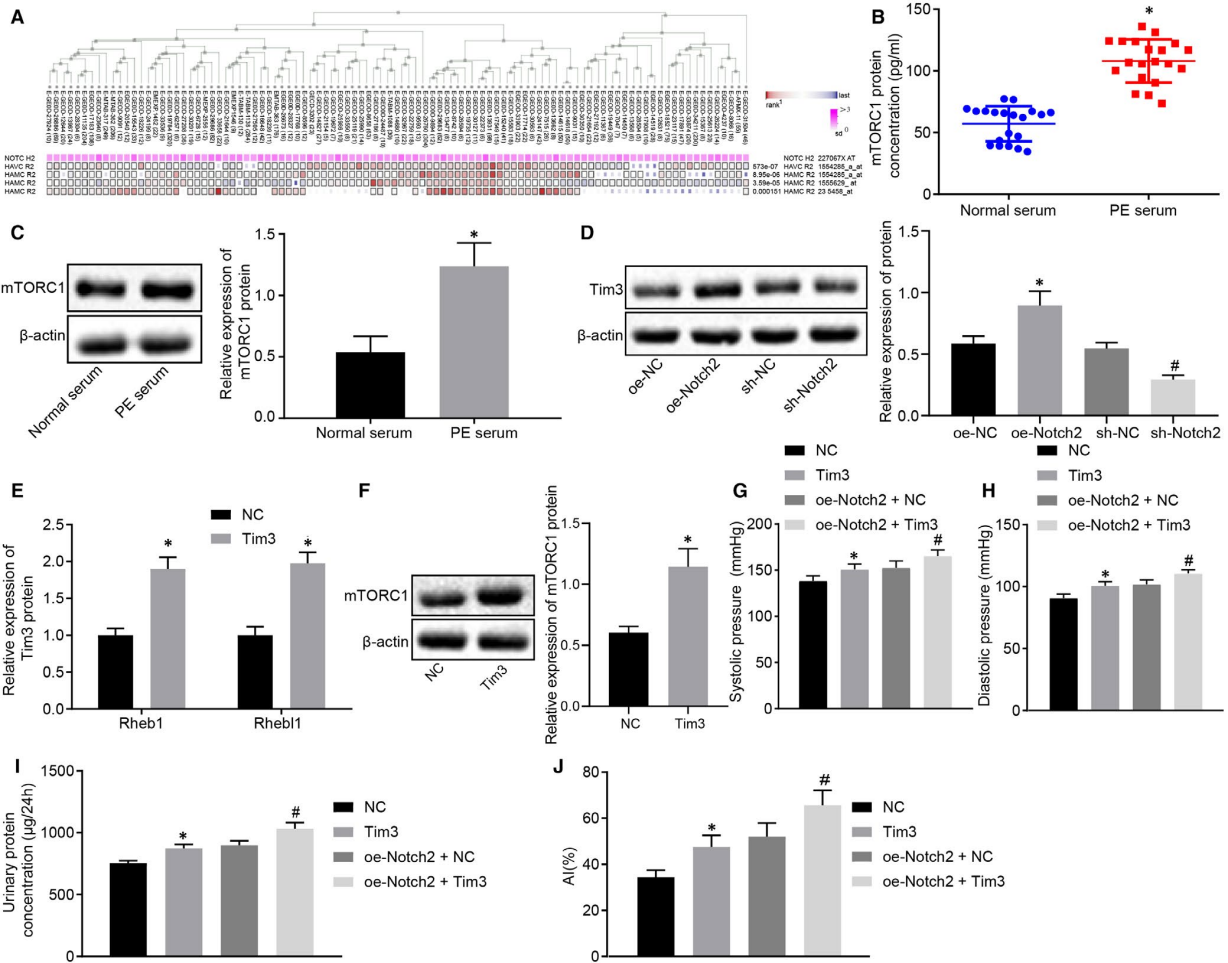
Notch2 worsens PE by activating the TIM3/mTORC1 signalling pathway. A, The co‐expression relationship between Notch2 and TIM3 predicted using MEM (*P* = 2.73E − 07). B, The expression of mTORC1 in the PE serum group or normal serum group determined by ELISA (n = 20). C, The protein expression of mTORC1 in the PE patients or normal placental tissues determined by immunoblotting. D, The expression of TIM3 in rats determined by immunoblotting (n = 5/group). E, The expression of mTORC1 activating factor Rheb1 and its isomer Rhebl1 in rats determined using RT‐qPCR. F, mTORC1 expression normalized to β‐actin in rats determined by Western blot analysis. G, The systolic blood pressure of rats. H, The diastolic blood pressure of rats. I, The 24‐h urinary protein concentration of rats. J, The cell apoptotic rate in placental tissues determined using TUNEL (×400). *P < .05 vs the normal serum, normal placental tissues, NC, oe‐NC groups; #*P* < .05 vs the sh‐NC or oe‐Notch2 + NC groups. The independent sample *t* test was conducted for the comparison of normally distributed data between two groups. Comparisons among multiple groups were conducted using one‐way ANOVA, followed by Turkey's post hoc test. Variables were analysed at different time points using Bonferroni‐corrected repeated measures ANOVA

Online analysis MEM identified that Notch2 and TIM3 had a significant co‐expression relationship (Figure 7A). To study the effect of Notch2 on PE symptoms, we treated PE model rats with corresponding plasmids. Initially, the mTORC1 concentration in the PE and normal serum was measured by ELISA (Figure 7B), which revealed reduced concentration of mTORC1 in the normal serum group, whereas opposite effects were seen for mTORC1 concentration in the PE serum group. Immunoblotting results (Figure 7C) revealed that compared with the normal placental tissues, the protein expression of mTORC1 in the PE patient tissues was potently increased. Immunoblotting (Figure 7D) to determine expression of TIM3 in rats displayed that compared with the oe‐NC group, rats in the oe‐Notch2 group exhibited marked up‐regulated level of TIM3, but an opposite effects were observed in the sh‐Notch2 group compared with the sh‐NC group.

RT‐qPCR and Western blot analysis results demonstrated that (Figure 7E,F) the expression of mTORC1 activating factor Rheb1 and its isomer Rhebl1 strikingly declined in the TIM3 group compared to the NC group and that mTORC1 expression was significantly elevated when TIM3 was overexpressed. Results displayed that (Figure 7G‐J) the systolic blood pressure, diastolic blood pressure, 24‐hour urinary protein concentration, apoptotic rate were potently elevated in the TIM3 or oe‐Notch + TIM3 groups compared to the NC or oe‐Notch + NC groups. Coherently, Notch2 exacerbated PE by activating the TIM3/mTORC1 signalling pathway.

## 4 DISCUSSION

Preeclampsia is a systemic syndrome occurring in 3%‐5% of pregnant women, which is responsible for considerable maternal and neonatal morbidity and mortality, despite aggressive treatment.^15^ PE is proposed to be a consequence of insufficient trophoblast invasion, inadequate vascular remodelling and imbalanced immune response.^16^ Thus, we aimed in the current study to explore how EV‐derived miR‐18b derived from HUCMSCs affects proliferation and invasion of trophoblasts in vitro and the damage in a rat model of PE in vivo. Returning to the hypothesis posed at the beginning of this study, we can confirm that EV‐encapsulated miR‐18b promotes trophoblast proliferation and invasion via the Notch2/TIM3/mTORC1 axis, thereby conferring protection against PE.

Initially, we found that HUCMSC‐encapsulated EVs promoted the trophoblast proliferation and invasion in vitro and ameliorated PE in vivo. Consistently, another study has noted that EVs derived from HUCMSCs could protect the morphology and angiogenesis of placenta in rats with PE.^5^ In the subsequent experiments, we also displayed that miR‐18b is poorly expressed in the placental tissues and HUCMSC‐derived EVs from PE patients and that EV‐derived miR‐18b from HUCMSCs enhanced the proliferation and invasion of trophoblasts. Concordant with our study results, miR‐18 expression was down‐regulated in the plasma and placenta of patients with PE.^17^ Conversely, the elevation of miR‐18b expression is reported to inhibit the invasion, migration of trophoblasts in PE.^8^ In addition, the elevation of EV‐derived miR‐18b contributed to decreased systolic blood pressure, diastolic blood pressure and 24‐hour urinary protein in a rat model of PE. Conversely, the systolic blood pressure, diastolic blood pressure and urinary protein were elevated in PE patients compared with findings in normal pregnancies.^18^ Therefore, we conclude that EV‐derived miR‐18b confers protection against PE in vivo.

Moreover, we demonstrated that Notch2 was negatively targeted by EV‐derived miR‐18b and that Notch2 down‐regulation promoted proliferation and invasion of trophoblasts. Consistently, a prior study noted that Notch2 expression is elevated in trophoblasts and that its suppression promoted proliferation and invasion of the percentage of trophoblasts in PE.^10^ Notch2 was identified to be targeted by miR‐18b via our bioinformatics analyses, although this association is scantly documented in the previous literature.

Subsequently, our study revealed that Notch2 exacerbated PE by activating the TIM3/mTORC1 axis, while TIM3 and mTORC1 levels were up‐regulated in the serum of PE patients. It has been previously proposed that TIM3 is positively targeted by Notch2.^11^ Furthermore, TIM3 expression was elevated in decidual tissues of PE compared with normotensive pregnancy and promoted the pathogenesis of PE.^13^ Interestingly, TIM3 has been reported to activate mTORC1, thus affecting T‐cell differentiation during Ag stimulation.^19^ The level of mTORC1 is thought to be up‐regulated in PE patients, and higher levels of mTORC1 would be consistent with the increased systolic pressure in that disorder.^20^ Furthermore, we also demonstrated that Notch2 exacerbated PE and that elevation of EV‐derived miR‐18b contributed to increased systolic blood pressure, diastolic blood pressure and 24‐hour urinary protein in our model by activating TIM3/mTORC1 axis in vivo.

In conclusion, the current study sheds light on the mechanism underlying the effect of EV‐derived miR from HUCMSCs on PE. Particularly, EV‐derived miR‐18b could down‐regulate Notch2 expression, thereby knocking down the expression of TIM3/mTORC1 axis. This mechanism might enhance the proliferation and invasion activity of trophoblasts in PE, which also introduces EV‐derived miR‐18b as a potential therapeutic target for PE. However, we only adopted a single dose of EVs in our experiments. Further studies should be aimed at identifying the optimum dose and the times of injection, and the regulatory role of miR‐18b and Notch2 in PE requires further validation.

## CONFLICT OF INTEREST

The authors declare that they have no conflicts of interests.

## AUTHOR CONTRIBUTIONS

**Zhongmei Yang:** Conceptualization (equal); data curation (equal); validation (equal); writing‐review and editing (equal). **Nan Shan:** Data curation (equal); formal analysis (equal); visualization (equal); writing‐review and editing (equal). **Qinyin Deng:** Formal analysis (equal); methodology (equal); validation (equal); writing‐review and editing (equal). **Yujue Wang:** Investigation (equal); methodology (equal); software (equal); supervision (equal); writing‐review and editing (equal). **Yan Hou:** Investigation (equal); supervision (equal); writing‐original draft (equal). **Jie Mei:** Conceptualization (equal); funding acquisition (equal); resources (equal); writing‐original draft (equal). **Zhao Wu:** Conceptualization (equal); funding acquisition (equal); methodology (equal); resources (equal); writing‐review and editing (equal).

## Supporting information

Fig S1

## Data Availability

The data that support the findings of this study are available from the corresponding author upon reasonable request.
